# 

**Published:** 1891-02

**Authors:** 


### BOOKS RECEIVED.

ESSENTIALS OF PRACTICE OF MEDICINE. By Henry Morris, M. D. W. B. Saunders, Medical Publisher, 913 Walnut St., Philadelphia, Pa.

Volume II.—Foster's Encyclopædic Medical Dictionary, by D. A. Appleton & Co., New York, N. Y.

Transactions of the American Orthopedic Association.

Prof. R. Koch's method to cure Tuberculosis, by H. E. Haferkorn, 367 Fourth Avenue, Milwaukee, Wis.

